# Regional Difference in Sex Steroid Action on Formation of Morphological Sex Differences in the Anteroventral Periventricular Nucleus and Principal Nucleus of the Bed Nucleus of the Stria Terminalis

**DOI:** 10.1371/journal.pone.0112616

**Published:** 2014-11-14

**Authors:** Moeko Kanaya, Mumeko C. Tsuda, Shoko Sagoshi, Kazuyo Nagata, Chihiro Morimoto, Chaw Kyi Tha Thu, Katsumi Toda, Shigeaki Kato, Sonoko Ogawa, Shinji Tsukahara

**Affiliations:** 1 Division of Life Science, Graduate School of Science and Engineering, Saitama University, Saitama, Japan; 2 Laboratory of Behavioral Neuroendocrinology, University of Tsukuba, Tsukuba, Japan; 3 Department of Biochemistry, School of Medicine, Kochi University, Nankoku, Japan; 4 Soma Central Hospital, Fukushima, Japan; John Hopkins University School of Medicine, United States of America

## Abstract

Sex steroid action is critical to form sexually dimorphic nuclei, although it is not fully understood. We previously reported that masculinization of the principal nucleus of the bed nucleus of the stria terminalis (BNSTp), which is larger and has more neurons in males than in females, involves aromatized testosterone that acts via estrogen receptor-α (ERα), but not estrogen receptor-β (ERβ). Here, we examined sex steroid action on the formation of the anteroventral periventricular nucleus (AVPV) that is larger and has more neurons in females. Morphometrical analysis of transgenic mice lacking aromatase, ERα, or ERβ genes revealed that the volume and neuron number of the male AVPV were significantly increased by deletion of aromatase and ERα genes, but not the ERβ gene. We further examined the AVPV and BNSTp of androgen receptor knockout (ARKO) mice. The volume and neuron number of the male BNSTp were smaller in ARKO mice than those in wild-type mice, while no significant effect of ARKO was found on the AVPV and female BNSTp. We also examined aromatase, ERα, and AR mRNA levels in the AVPV and BNSTp of wild-type and ARKO mice on embryonic day (ED) 18 and postnatal day (PD) 4. AR mRNA in the BNSTp and AVPV of wild-type mice was not expressed on ED18 and emerged on PD4. In the AVPV, the aromatase mRNA level was higher on ED18, although the ERα mRNA level was higher on PD4 without any effect of AR gene deletion. Aromatase and ERα mRNA levels in the male BNSTp were significantly increased on PD4 by AR gene deletion. These results suggest that estradiol signaling via ERα during the perinatal period and testosterone signaling via AR during the postnatal period are required for masculinization of the BNSTp, whereas the former is sufficient to defeminize the AVPV.

## Introduction

Morphological sex differences in the brain are responsible for sex differences in brain functions. The bed nucleus of the stria terminalis (BNST) in the forebrain contains a subnucleus that shows morphological sex differences. This subnucleus of the BNST is called the principal nucleus of the BNST (BNSTp). In rodents, the BNSTp of males is larger and has more neurons than that of females [1], [2], and it is involved in the regulation of male sexual behavior [3], [4], [5]. Males have a larger number of arginine vasopressin neurons in the BNSTp than in females [6]. This anatomical sexual difference is related to sex differences in the expression of aggressive behaviors [7].

The anteroventral periventricular nucleus (AVPV) in female rodents is larger and has a higher cell density than that in males [8], [9], [10]. The female AVPV contains many more neurons expressing tyrosine hydroxylase (TH) [11] and kisspeptin [12], [13] than those in the male AVPV. The AVPV plays an essential role in controlling the luteinizing hormone surge that induces ovulation in female rodents [14]. Specifically, kisspeptin neurons in the female AVPV mediate the positive-feedback action of estradiol that stimulates gonadotropin-releasing hormone secretion, followed by induction of the luteinizing hormone surge [15].

Morphological sex differences in the rodent brain are formed under the influence of sex-biasing factors which are the effects of sex chromosome genes expressed outside of the gonads and the effects of gonadal steroids during the perinatal period and pubertal/adolescent periods [16], [17], [18], [19]. Of these sex-biasing factors, organizational effects of testicular testosterone during the perinatal period have been studied extensively. However, the mechanism of action remains to be determined fully. In rodents, testosterone secreted from the testes during the perinatal period and converted to estradiol by aromatase acts to organize male-typical structures in the brain [20]. Neonatal castration of male rats decreases the volume and neuron number of the BNSTp in adulthood, whereas administration of testosterone propionate (TP) to newborn females increases them [21]. Administration of estradiol benzoate (EB) to newborn female mice also increases the neuron number of the BNSTp in adult females [22]. We previously reported that masculinization of the BNSTp is disrupted by deletion of aromatase or estrogen receptor-α (ERα) genes, but not the estrogen receptor-β (ERβ) gene [23]. This finding suggests that masculinization of the BNSTp involves the action of aromatized testosterone that signals via ERα rather than ERβ. However, this action may be not sufficient for masculinization of the BNSTp, because the BNSTp volume in female mice is not completely masculinized by postnatal EB treatment. On the other hand, such treatment is effective to increase the neuron number of the female BNSTp to that of the male BNSTp [22], [23]. Testicular feminization mutation (tfm), which renders the androgen receptor (AR) less functional, reduces the volume [24] and number of arginine vasopressin neurons [25] in the BNSTp of male rats. Thus, the actions of both testosterone via binding to AR and aromatized testosterone via binding to ERα are required for masculinization of the BNSTp.

Testicular testosterone during the perinatal period is also important to form male-typical structures in the AVPV, but its effects are contradictory to those on the male BNSTp. Administration of TP to postnatal female rats reduces the AVPV volume and the numbers of TH-expressing neurons and kisspeptin neurons in the AVPV [12], [25]. The AVPV volume in female guinea pigs is also decreased by prenatal TP treatment [26]. The number of TH-expressing neurons in the AVPV of male mice is increased by deletion of ERα or ERβ genes [27], [28]. Neonatal treatment with agonists selective for either ERα or ERβ decreases the neuron number of the AVPV in female rats [29]. However, the number of TH-expressing neurons in the AVPV of tfm male mice is comparable to that in normal male mice [27]. Therefore, AVPV is probably defeminized by the actions of testosterone that is converted to estradiol by aromatase when this androgen-derived estrogen binds to ERα and ERβ.

A lack of functional AR in tfm decreases the BNSTp volume and the number of arginine vasopressin neurons in the BNSTp of male rats [24], [25], but it does not disrupt the sex difference in the number of TH-expressing neurons in the AVPV of mice [27]. These findings indicate that there may be a regional difference in the actions of androgens on the formation of sexually dimorphic nuclei. However, such a difference has not been documented definitively because the endpoints and animal species used for these analyses differ among studies. In this study, we aimed to determine the mechanisms of sex-steroid action on sexual differentiation of the AVPV and BNSTp, and the differences between the AVPV and BNSTp with regard to these mechanisms. We examined the morphology of the AVPV in aromatase knockout (AromKO), ERα knockout (αERKO), and ERβ knockout (βERKO) mice. We also examined the morphology of the AVPV and BNSTp in AR knockout (ARKO) mice. We then measured the levels of aromatase, ERα, ERβ, and AR mRNAs in the AVPV and BNSTp of wild-type (WT) and ARKO mice during the perinatal period to determine the influence of age and sex on these gene expression and the effects of AR gene deletion.

## Materials and Methods

### Animals

Adult AromKO [30], αERKO [31], and βERKO [32] mice and their respective WT littermates were obtained from litters resulting from mating between heterozygous mice derived from the respective breeding colonies. ARKO mice [33] and their WT littermates were obtained from litters resulting from mating between male mice with a floxed AR gene and heterozygous ARKO female mice expressing Cre recombinase. Mice from the breeding colonies were all completely backcrossed to C57BL/6J mice. For genotyping, PCR amplification of genomic DNA extracted from tail tissue was performed to identify AromKO, αERKO, βERKO, and ARKO, and respective WT mice (see “Genotyping and selection of animals used for experiments” for details).

All mice were housed in a room maintained at 22°C with a 12 h light/12 h dark cycle (lights on at 08:00 h) with free access to a standard diet and tap water. All animal procedures were approved and performed in accordance with the Guidelines for the Care and Use of Experimental Animals of the University of Tsukuba and Saitama University.

### Genotyping and selection of animals used for experiments

Male and female heterozygous AromKO, αERKO, and βERKO mice were mated to obtain offspring. Genotypes of the offspring were determined by PCR amplification of tail DNA as described previously [30], [31], [32]. The primer sequences used for genotyping and amplicon sizes are shown in Table 1. Homozygous knockout mice of aromatase, ERα, or ERβ genes, and their respective WT littermates were used for experiments.

**Table 1:** Sequences of primers used for genomic PCR.

Transgenic mice	Forward primer sequence (5′ to 3′)	Reverse primer sequence (5′ to 3′)	Amplicon size (bp)
AromKO	GTGACAGAGACATAAAGATCGAGGAT	AAATTCATTGGGCTTAGGGAAGTACTCG	218 for WT
	GTGACAGAGACATAAAGATCGAGGAT	TCAGAGCAGCCGATTGTCTGTTGTGCCCAGTCAT	410 for AromKO
αERKO	CGGTCTACGGCCAGTCGGGCATC	CAGGCCTTACACAGCGCCACCC	400 for WT
	CGGTCTACGGCCAGTCGGGCATC	GCTGACCGCTTCCTCGTGCTTTAC	750 for αERKO
βERKO	TGGACTCACCACGTAGGCTC	CATCCTTCACAGGACCAGACAC	450 for WT
	TGGACTCACCACGTAGGCTC	GCAGCCTCTGTTCCACATACAC	500 for βERKO
ARKO	CATTTGGGCCAGCTAAACAT	ATTCTCCCACCGTCAGTACG	308 for Cre
	AAGATAAGCTTACATAATCACATGGA	CCTATGAAATCCTTTGCTGCACATGT	814 for AR
	AAGATAAGCTTACATAATCACATGGA	CCTATGAAATCCTTTGCTGCACATGT	629 for ZFY-1
	AAGTGAATGGTCTTGGC	TTACAGGTCTGGTGCAAGCC	400 or 430 for AR or floxed AR

Transgenic male mice with their AR gene flanked by lox P sequences on the X chromosome were mated with heterozygous ARKO females expressing Cre recombinase under the control of cytomegalovirus promoter. Targeted disruption of the AR gene was then performed by the Cre-lox P system as described previously [34]. Offspring with four different genotypes in each sex were generated as follows: Cre AR^-/Y^ (CreKO), Cre AR^+/Y^ (CreWT), AR^-/Y^ (KO), and AR^+/Y^ (WT) in male offspring, and Cre AR^-/-^ (CreKO), Cre AR^-/+^ (CreHZ), AR^flox/-^ (FloxHZ), and AR^flox/+^ (FloxWT) in female offspring (see Figure S1). To identify the genotype and sex of the offspring, we performed two rounds of genomic PCR. First, multiplex genomic PCR was performed using tail DNA to amplify *Cre*, *AR*, and *ZFY-1*, which is located on Y chromosome and encodes a zinc finger transcription factor [35]. The first multiplex genomic PCR resulted in determination of four genotypes of male offspring (CreKO, CreWT, KO, and WT males), two genotypes of female offspring (CreKO and CreHZ females), and one group consisting of two genotypes of female offspring (FloxHZ and FloxWT females). Next, to discriminate FloxHZ from FloxWT in females, genomic PCR was carried out using the tail DNA of female offspring that were determined as either FloxHZ or FloxWT by the first genomic PCR. The primer sequences and amplicon sizes are summarized in Table 1.

In this study, we used CreWT, CreKO, WT, and KO males. We performed morphometrical analysis of the AVPV and BNSTp in these animals. As a result, the CreWT and WT males did not differ significantly with regard to the volume of the AVPV or BNSTp and the numbers of neuronal and glial cells in the AVPV or BNSTp. Similarly, CreKO and KO males did not differ significantly in these aspects (data not shown). Accordingly, no significant effect of Cre was found on the morphology of the AVPV and BNSTp. Therefore, CreWT and WT males were designated as WT males, and CreKO and KO males were designated as ARKO males (see Figure S1). We used FloxWT females as the WT females and CreKO females as ARKO females in this study.

### Experimental Design

#### Experiment 1: Morphometrical analysis of the AVPV in adult AromKO, αERKO, βERKO, and ARKO mice

The morphology of the AVPV in adult AromKO, αERKO, βERKO, and ARKO mice and their adult WT littermates of both sexes (age: 15–30 weeks old, n = 5 for AromKO mice and their WT littermates of each sex; age: 12–34 weeks old, n = 3–5 for αERKO mice and their WT littermates of each sex; age: 15–24 weeks old, n = 5 for βERKO mice and their WT littermates of each sex; age: 11–26 weeks old; n = 4–10 for ARKO mice and their WT littermates of each sex) was examined to determine the roles of aromatase, ERα, ERβ, and AR in sexual differentiation of the AVPV. After preparation of coronal brain sections from these animals, a stereological method was used to estimate the volume of the AVPV and the numbers of neuronal and glial cells in the AVPV (see “Morphometrical analysis” for details).

#### Experiment 2: Morphometrical analysis of the BNSTp in adult ARKO mice

Morphometrical analysis of the BNSTp in adult ARKO mice was performed to determine the effects of a null AR mutation on sexual differentiation of the BNSTp. For both sexes, coronal sections of brains obtained from adult ARKO mice and their adult WT littermates (age: 11–26 weeks old; n = 4–10 for each genotype and sex) were used for stereological analysis to estimate the volume and the numbers of neuronal and glial cells in the BNSTp (see “Morphometrical analysis” for details).

Serum testosterone levels in newborn ARKO male mice tend to be lower than those in newborn WT males, although it is not significantly different [34]. To exclude the possibility that the phenotype of developing ARKO male mice is due to decreased testosterone production rather than the null AR mutation, we injected TP (100 µg dissolved in 20 µL sesame oil) or the vehicle only subcutaneously into WT and ARKO male mice on the day of birth (n = 4–6 for each genotype and treatment). After sexual maturation (8–11 weeks old), a stereological method was used to estimate the volume and the numbers of neuronal and glial cells in the BNSTp of each animal (see “Morphometrical analysis” for details).

#### Experiment 3: Analysis of gene expression in the AVPV and BNSTp of perinatal WT and ARKO mice

We measured the mRNA levels of AR, aromatase, ERα, and ERβ in the AVPV and BNSTp from male and female WT littermates of ARKO mice on embryonic day (ED) 18 and postnatal day (PD) 4 to determine the mechanism responsible for sex steroid actions on the AVPV and BNSTp during the perinatal period (n = 3–6 for each genotype, sex, and age). The day of vaginal plug confirmation was defined as ED1, and the day of birth was defined as PD0. The ages of animals for brain sampling were in accordance with a report showing the temporal changes in testicular testosterone production of perinatal mice, in which testosterone production reached to a peak level on ED18 and then declined after birth followed by an increase on PD4 [36]. We also measured aromatase, ERα, and ERβ mRNA levels in the AVPV and BNSTp of female and male ARKO mice on ED18 and PD4 to examine the effects of AR gene deletion.

The laser microdissection method was used to remove tissue fragments of the AVPV and BNSTp from coronal brain sections (see “Gene expression analysis” for details). Total RNA was extracted from the tissue fragments and quantitated, and equal amounts of total RNA (approximately 130 µg) were used for first-strand cDNA synthesis via reverse transcription. cDNA samples were then applied to real-time PCR analyses to measure the mRNA levels of AR, aromatase, ERα, and ERβ. The mRNA levels of target genes were normalized to the amount of total RNA.

### General procedures

#### Morphometrical analysis

Adult mice were deeply anesthetized with sodium pentobarbital (60 mg/kg body weight) and then intracardially perfused with 0.1 M phosphate-buffered saline (PBS; pH 7.5), followed by 4% paraformaldehyde in 0.1 M phosphate buffer (PB; pH 7.5). Their brains were removed and postfixed with the same fixative at 4°C overnight and then immersed in 30% sucrose in 0.1 M PB for 2 days at 4°C. Brains were coronally sectioned at a thickness of 30 µm by a cryostat at 60 µm intervals. The brain sections were stained with a 0.1% cresyl fast violet solution and then used for morphometrical analysis.

We estimated the volume of the AVPV and BNSTp, and total numbers of neuronal and glial cells in both nuclei with the aid of Stereo Investigator software (MBF Bioscience, Inc., Williston, VT, USA). The identification number of each animal was encoded to ensure that the person who performed the analysis was blinded to the source of each sample. We used the optical fractionator method in accordance with the system workflow of Stereo Investigator. We first traced the outlines of AVPV and BNSTp regions on the left side of each brain section to calculate the volume and select the area of analysis. To analyze the AVPV, we used three to five brain sections (see Tables S1–S4 for section numbers in each group) among which the section containing the rostral edge of the internal capsule was selected as the first section at the anteroposterior level. To analyze the BNSTp, we used three to seven brain sections (see Tables S5 and S6 for section numbers in each group) among which the first section at the anteroposterior level was the section that was the next to the section containing the caudal edge of the anterior part of the anterior commissure, and the last section was before the section with the dorsal and ventral parts of the stria medullaris. We then counted cells, which can be potentially identified as neuronal or glial cells by staining with cresyl fast violet, in the AVPV and BNSTp using the procedure in our previous study [23]. In this study, cells were defined as neuronal cells when their cytoplasm was stained with cresyl fast violet and the cell bodies contained an oval or spherical nucleus with a clear nucleolus, while cells were defined as glial cells when their nucleus was smaller than that of neuronal cells and were densely stained with cresyl fast violet. Cells were excluded when they were close to blood capillaries and potentially endothelial cells (see Figure S2). Detailed parameters of the stereological analysis are summarized in Tables S1–S6. In addition to semi-quantification of cell number, we calculated the density of neuronal and glial cells in the AVPV and BNSTp by dividing the estimated cell number by the volume of the nucleus.

#### Gene expression analysis

ED18 fetal mice were obtained from pregnant dams via Caesarean section after pregnant mothers were deeply anesthetized by intraperitoneal injection of sodium pentobarbital (50 mg/kg body weight). ED18 and PD4 mice were decapitated and their brain tissues were immediately frozen and stored at −80°C until further processing.

Brains were coronally sectioned at a thickness of 25 µm for ED18 mice and 30 µm for PD4 mice. The sections were mounted on PEN-membrane slides (Leica Microsystems, Wetzlar, Germany) and immediately fixed with ice-cold 5% acetic acid in ethanol for 3 min. After rinsing in ice-cold RNase-free water for 1 min, the sections were stained with ice-cold 0.2% cresyl fast violet for 1 min. The sections were rinsed twice in ice-cold RNase-free water for 1 min each time and then left to dry in cool air. A laser microdissection system (Leica LMD 7000; Leica Microsystems) was used to dissect out AVPV and BNSTp tissues from the cresyl fast violet-stained sections. The borders of the AVPV and BNSTp were irradiated with a pulsed UV laser beam to cut the nuclei from the sections. The isolated section fragments for each nucleus were collected in a PCR tube containing 70 µL RNA extraction buffer from a RNeasy Micro Kit (Qiagen, Valencia, CA, USA). Samples were stored at −80°C until further processing.

Total RNA was extracted and purified using the RNeasy Micro Kit according to the manufacturer's protocol. The RNA concentration was measured by a spectrophotometer (BioPhotometer plus, Eppendorf, Germany; TrayCell, Hellma, USA). First-strand cDNA was synthesized using a TaKaRa Prime Script RT reagent kit (TaKaRa Bio, Inc., Otsu, Japan) according to the manufacturer's protocol. For each sample, total RNA (approximately 130 ng) was reverse transcribed to first-strand cDNA in a final volume of 15 µL Prime Script buffer containing Prime Script RT Enzyme Mix I (0.75 µL) and random hexamers (75 pmol). Standardized samples for aromatase, ERα, and ERβ were prepared by mixing an equal amount of each unknown cDNA sample and then serially diluting in EASY Dilution (TaKaRa Bio) (×1, ×2, ×4, ×8, ×16, and ×32). To prepare standardized samples for AR, cDNA was synthesized from total RNA isolated from the hypothalamus of adult WT mice and then serially diluted at ×8, ×16, ×32, ×64, ×128, ×256, and ×512. Real-time PCR analysis was performed using a Light Cycler ST300 (Roche Diagnostics, Mannheim, Germany) with the method described in our previous studies [37], [38]. Two microliters of standardized or unknown samples were amplified in a 20 µL reaction mixture containing 200 nM of each gene-specific primer (see Table 2) and 10 µL of 2× SYBR Premix Ex Taq (TaKaRa Bio).

**Table 2:** Sequences of primers used for real-time PCR.

Target gene	Forward primer sequence (5′ to 3′)	Reverse primer sequence (5′ to 3′)	Amplicon size (bp)	NCBI reference sequence
AR	AAGCTGAAGAAACTTGGAAATCTAAA	TCAATGGCTTCCAGGACG	155	NM_013476
Aromatase	GAGGATGACGTAATTGACGG	AAAGGCTGAAAGTACCTGTAG	155	NM_007810.3
ERα	GTTGAAGTTTAATTGCTTGTTTATTGGAC	CATGACACGGTAGTTTGAAACG	156	NM_007956.4
ERβ	CTGTGATGAACTACAGTGTTCCC	GGTTCTGCATAGAGAAGCGAT	151	NM_207707.1, NM_010157.3

Primers for ERβ were designed to amplify both ERβ1 and ERβ2 cDNAs.

#### Statistical analysis

Two-way analysis of variance (ANOVA) was used to determine the main effect of genotype and sex, and the interaction between genotype and sex on the volume of the AVPV or BNSTp and the numbers and densities of neuronal and glial cells in the AVPV or BNSTp. Three-way ANOVA was performed to determine the main effects of sex, genotype, and age and their two- or three-way interactions on the mRNA levels of aromatase, ERα, or ERβ. Tukey's test was used for post-hoc analysis when the effects of interactions among main factors were significant. The Student's *t*-test was applied to assess differences in the mRNA levels of AR in WT male and female mice on PD4 and those of aromatase, ERα, and ERβ in WT and ARKO mice on PD4 in each sex. For analyses using Tukey's test and the Student's *t*-test, p<0.05 was considered significant.

## Results

### Experiment 1

#### Effects of aromatase, ERα, ERβ, and AR gene deletions on the AVPV

**Figure 1:**
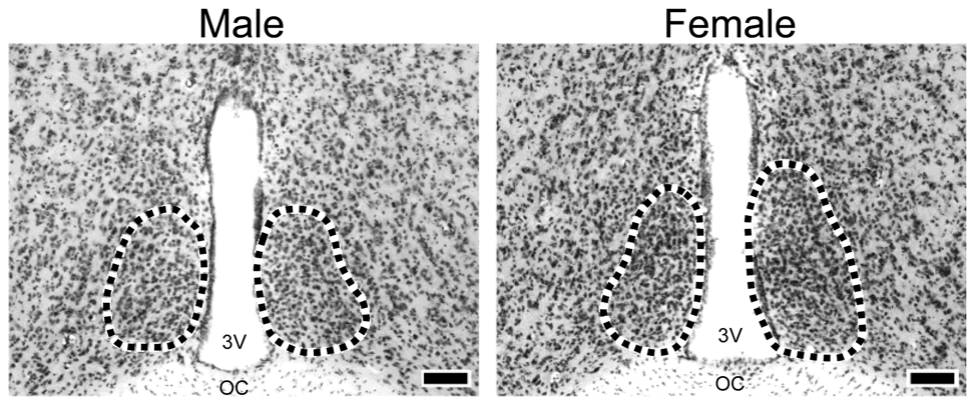
Representative photomicrographs of the AVPV in adult mice. Photomicrographs show WT male and female mice. Dotted lines indicate the border areas of the AVPV. Scale bars indicate 100 µm. oc, optic chiasm; 3V, third ventricle.

**Figure 2:**
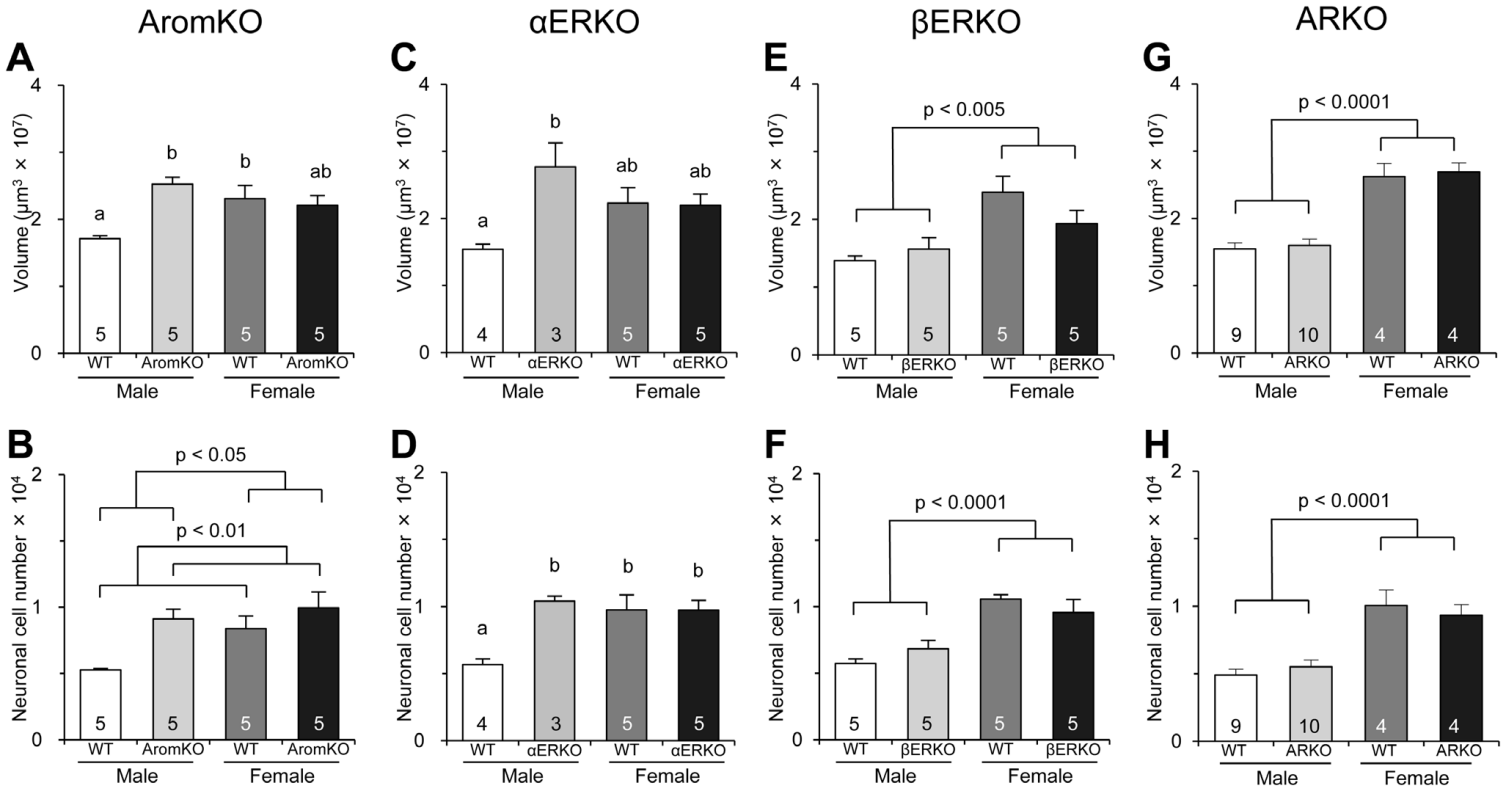
Effects of aromatase, ERα, ERβ, and AR gene deletions on the AVPV. The volume (**A, C, E, and G**) and neuronal cell number (**B, D, F, and H**) in the AVPV of WT, AromKO, αERKO, βERKO, and ARKO mice. The number in each column indicates the number of animals used in the experiments. Values are the mean ± SEM. Values with different letters differ significantly (p<0.05).

In WT littermates of transgenic mice lacking aromatase, ERα, ERβ, or AR genes, the AVPV was larger and had more neuronal cell bodies in female mice than that in male mice (Figure 1). The morphology of the AVPV in male mice, but not female mice, was affected by deletion of aromatase and ERα genes, whereas there was no significant effect of ERβ and AR gene deletions. The volume of the AVPV in AromKO males and the volume and neuron number of the AVPV in αERKO males were significantly greater than those in WT males (Figure 2A, 2C, and 2D). In addition, the neuron number of the male AVPV was increased in mice lacking the aromatase gene. The neuron number of the AVPV was significantly higher in AromKO mice than that in WT mice and higher in female mice than that in male mice (Figure 2B). The volume and neuron number of the AVPV were greater in female mice than those in male mice despite the lack of ERβ and AR genes (Figure 2E–2H). The density of AVPV neurons did not differ significantly between sexes and was unaffected by deletion of ERα, ERβ, or AR genes, although it was significantly higher in female mice and transgenic mice among AromKO and WT mice (Tables S1–S4). The number of glial cells in the AVPV was not altered by sex or deletion of ERα or ERβ genes (Tables S2 and S3). On the other hand, deletion of the aromatase gene resulted in a significant increase in the glial cell number of the AVPV (Table S1). The glial cell number in the AVPV was significantly higher in WT and ARKO female mice than that in WT and ARKO male mice (Table S4). The density of glial cells in the AVPV was not changed by sex or deletion of any gene.

### Experiment 2

#### Effects of AR gene deletion on the BNSTp

**Figure 3:**
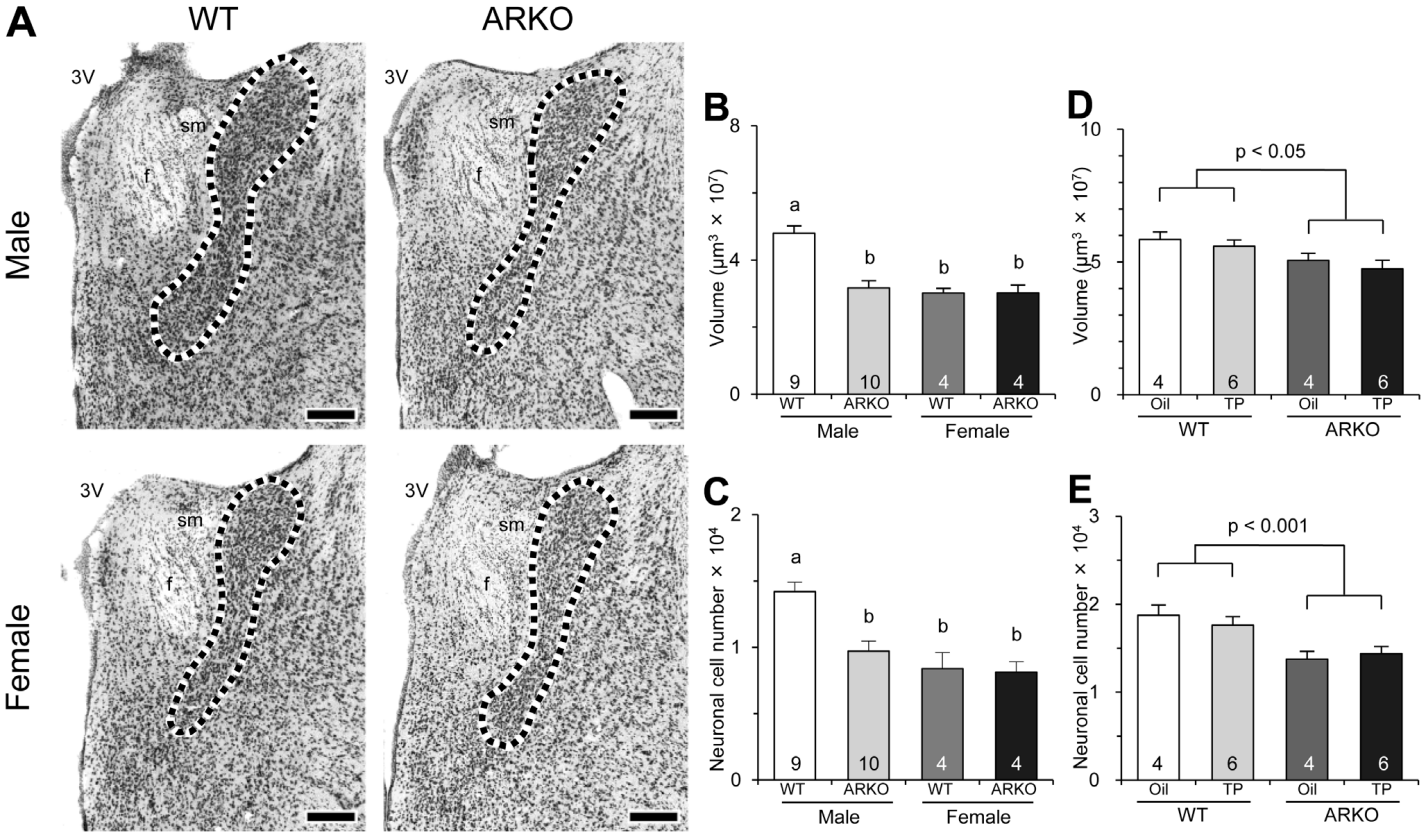
Effects of AR gene deletion on the BNSTp. Photomicrographs (**A**) show the BNSTp in WT and ARKO mice of both sexes. Dotted lines indicate border areas of the BNSTp. Scale bars indicate 200 µm. f, fornix; sm, stria medullaris of thalamus; 3V, third ventricle. The volume (**B**) and neuronal cell number (**C**) in the BNSTp of WT and ARKO mice. The volume (**D**) and neuronal cell number (**E**) in the BNSTp of WT and ARKO male mice that were treated with TP or sesame oil (vehicle) on the day of birth. See Figure 2 for details.

Although the morphology of the AVPV did not change in mice lacking the AR gene, the BNSTp was affected by AR gene deletion. The size of the BNSTp in ARKO male mice, but not ARKO female mice, was significantly smaller than that in WT littermates of the same sex (Figure 3A and 3B). The neuron number of the BNSTp in ARKO males was also significantly lower than that in WT males, and was comparable to that in WT and ARKO females (Figure 3C). The density of neurons and the number and density of glial cells in the BNSTp were not affected by deletion of the AR gene and did not differ between sexes (Table S5). Thus, morphometrical analyses of ARKO male mice indicated that there was a regional difference in the effects of the AR gene deletion between the AVPV and BNSTp. AR gene deletion increased the volume and neuron number of the male BNSTp, but did not affect those of the male AVPV.

#### Effects of neonatal TP treatment on the BNSTp of ARKO male mice

Neonatal TP treatment did not rescue the significant effects of AR gene deletion on the BNSTp in male mice. The volume and neuron number of the male BNSTp were significantly smaller in ARKO males than those in WT males with or without neonatal TP treatment (Figure 3D and 3E). The density of neuronal cells and the number and density of glial cells in the BNSTp were not changed by AR gene deletion or neonatal TP treatment (Table S6).

### Experiment 3

#### Isolation of the AVPV and BNSTp by laser microdissection

**Figure 4:**
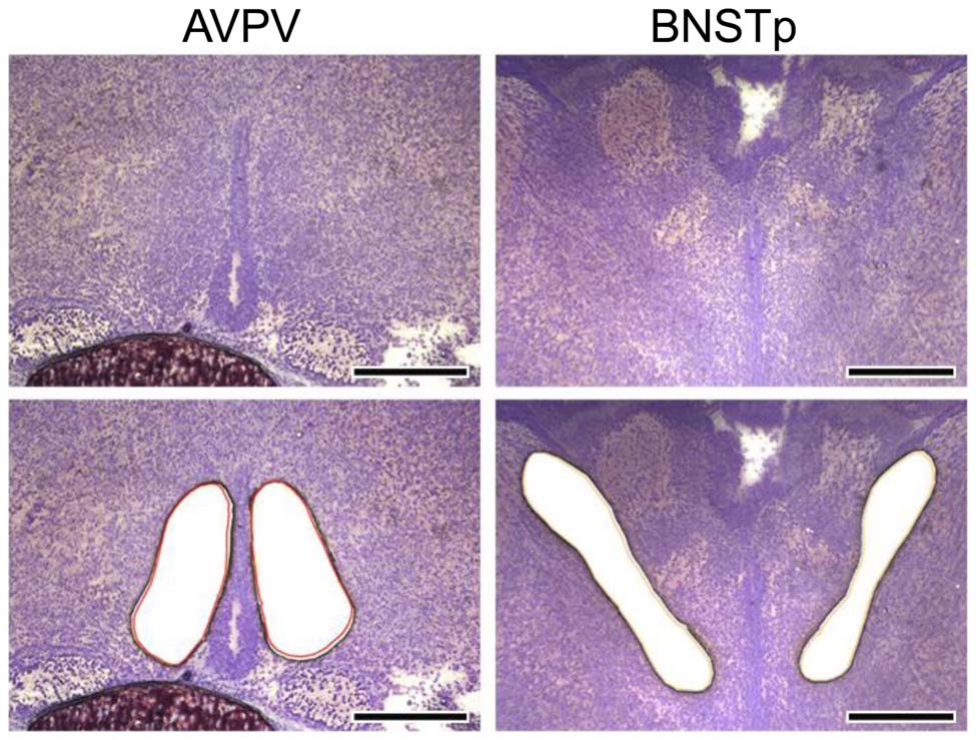
Representative photomicrographs of brain sections before and after isolation of the AVPV and BNSTp. Photomicrographs show coronal brain sections of ED18 mice. Scale bars indicate 200 µm in the AVPV and 300 µm in the BNSTp.

AVPV and BNSTp tissues were isolated from cresyl fast violet-stained brain sections of ARKO and WT mice on ED18 and PD4 by laser microdissection (Figure 4). There was no striking difference in the sizes of the isolated AVPV and BNSTp between ED18 and PD4.

#### Gene expression in the AVPV of ED18 and PD4 mice and effects of AR gene deletion

**Figure 5:**
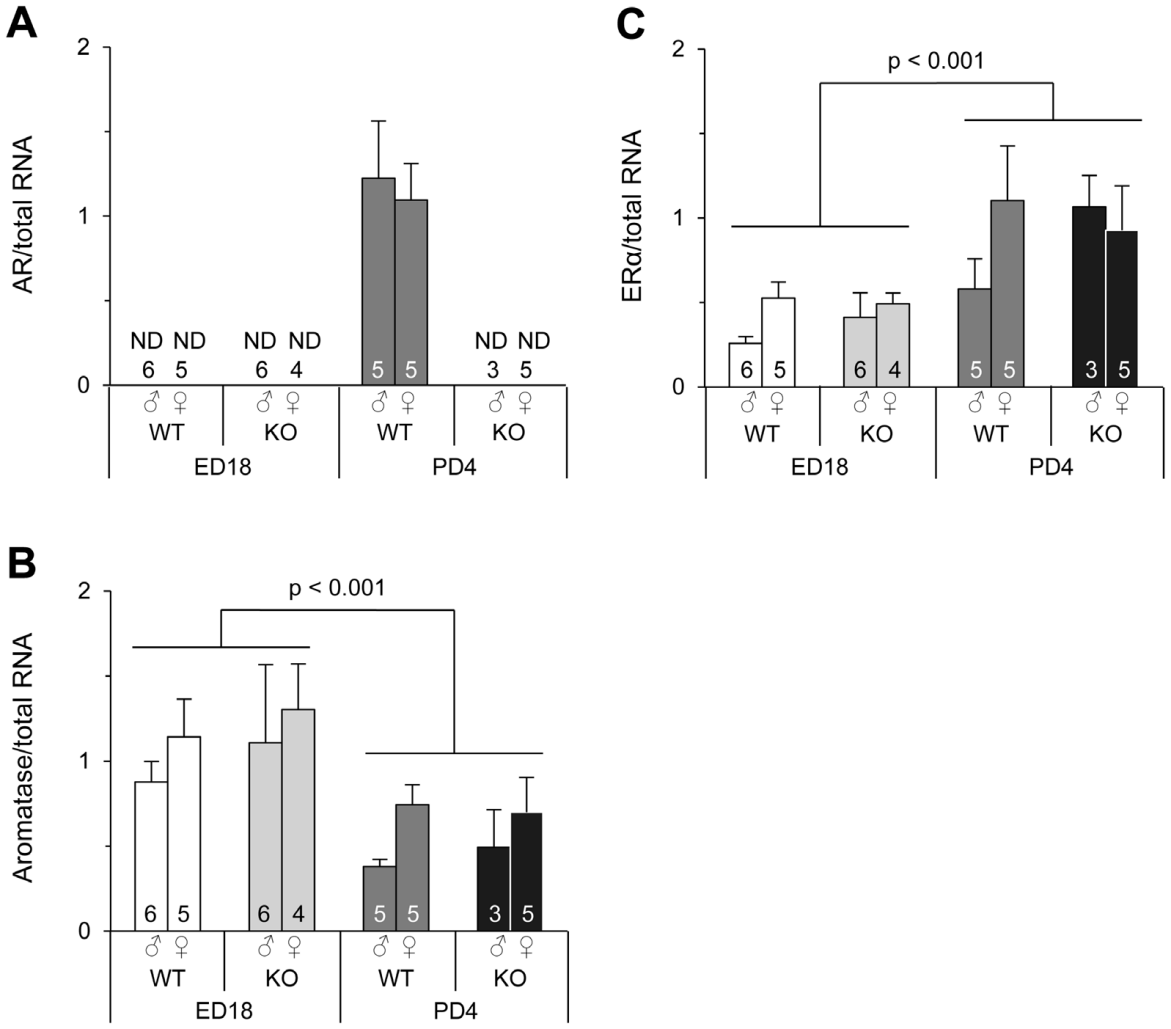
Gene expression in the AVPV of WT and ARKO mice on ED18 and PD4. The mRNA levels of AR (**A**), aromatase (**B**), and ERα (**C**) were normalized to the amount of total RNA. The number in each column indicates the number of animals used in the experiments. Values are the mean ± SEM. ND, not detected. AR mRNA levels in two of six WT males on ED18 were almost undetectable (data not shown).

As predicted, AR mRNA in the AVPV was undetectable in ARKO mice regardless of sex (Figure 5A). Although WT mice had the normal AR gene, AR mRNA levels in the AVPV were extremely low on ED18, and the AR mRNA levels in four of six males and five females were undetectable. The remaining two WT males on ED18 hardly expressed AR at detectable levels (data not shown). In contrast, AR mRNA was highly expressed in the AVPV of WT mice on PD4 without a sex difference. The mRNA levels of aromatase and ERα in the AVPV were different between ED18 and PD4. Aromatase mRNA levels in the AVPV were significantly higher on ED18 than those on PD4 (Figure 5B), whereas ERα mRNA levels were significantly higher on PD4 than those on ED18 (Figure 5C). Aromatase and ERα mRNA levels were not affected by deletion of the AR gene and similar in both sexes. ERβ mRNA levels in the AVPV of ED18 and PD4 mice were very low. The ERβ mRNA levels were undetectable in most samples. Detectable levels of ERβ mRNA were only found in one ARKO male on ED18, three ARKO males on PD4, and one ARKO female on PD4, but near the least detectable level of analysis in this study (data not shown).

#### Gene expression in the BNSTp of ED18 and PD4 mice and effects of AR gene deletion

**Figure 6:**
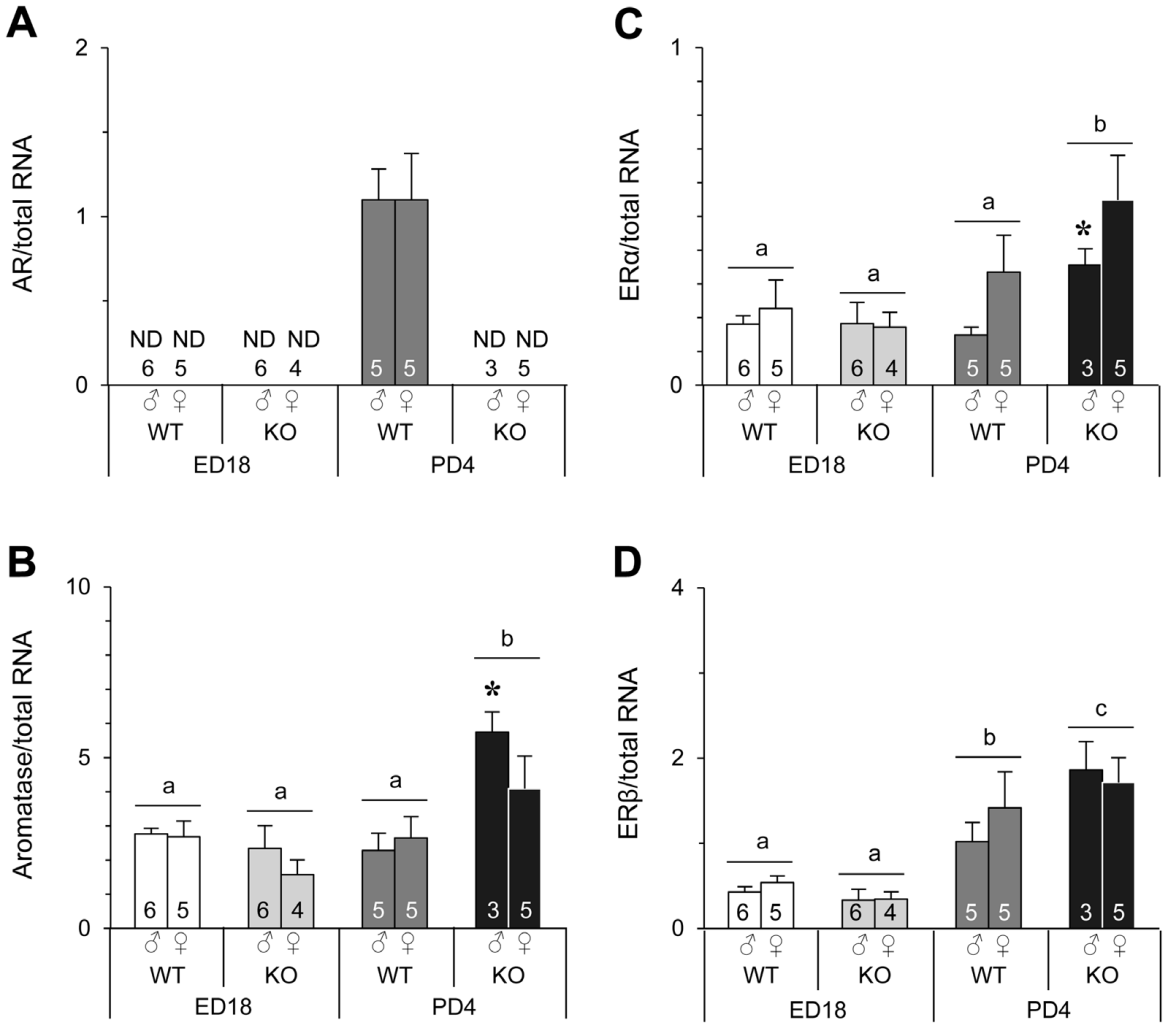
Gene expression in the BNSTp of WT and ARKO mice on ED18 and PD4. The mRNA levels of AR (**A**), aromatase (**B**), ERα (**C**), and ERβ (**D**) were normalized to the amount of total RNA. The number in each column indicates the number of animals used in the experiments. Values (mean ± SEM) with different letters differ significantly (p<0.05). *, p<0.05 vs. WT male mice on PD4. ND, not detected. AR mRNA levels in one WT male and one WT female were almost undetectable on ED18 (data not shown).

In addition to the AVPV of ARKO mice, the AR mRNA level was undetectable in the BNSTp of ARKO mice (Figure 6A). AR mRNA levels in the BNSTp of WT mice were also undetectable on ED18, except in one mouse for each sex, in which the AR mRNA level was close to the least detectable level (data not shown). Conversely, on PD4, the BNSTp of WT mice in both sexes expressed AR mRNA at a high level without a sex difference. In terms of aromatase, ERα, and ERβ, their mRNA expression was completely different between the BNSTp and AVPV in perinatal mice. In the BNSTp, aromatase mRNA levels were significantly increased on PD4 by AR gene deletion (Figure 6B). Specifically, when compared between WT and ARKO mice of each sex, aromatase mRNA levels in the BNSTp of ARKO male mice were significantly higher than those of WT male mice on PD4, but no significant difference was found between WT and ARKO female mice. Age-difference patterns and effects of AR gene deletion on ERα mRNA expression in the BNSTp were the same as those of aromatase mRNA expression. ERα mRNA levels in the BNSTp were significantly increased on PD4 by AR gene deletion, which was mainly due to a significant increase in ARKO male mice (Figure 6C). ERβ mRNA was detected in the BNSTp on ED18 and PD4, although it was not detected in the AVPV on the same days. The mRNA levels of ERβ in the BNSTp of WT and ARKO mice were significantly higher on PD4 than those on ED18 (Figure 6D). In addition, ERβ mRNA levels in the BNSTp on PD4 were significantly higher in ARKO mice than those in WT mice. Thus, the patterns of aromatase, ERα, and ERβ expression from ED18 to PD4 were different between the AVPV and BNSTp, and AR gene deletion caused significant changes in the expression of these genes in the BNSTp, but not the AVPV.

## Discussion

### Differences in sex steroid actions required for defeminization of the AVPV and masculinization of the BNSTp

In this study, we examined the morphology of the AVPV and BNSTp in transgenic mice lacking genes that are considered to participate in sexual differentiation of the brain. We found that the volume and neuron number of the AVPV in male mice, but not female mice, were increased by deletion of aromatase and ERα genes, although deletion of the ERβ gene had no effect. We previously reported that the volume and neuron number of the BNSTp in male mice are decreased by deletion of aromatase and ERα genes [23]. The findings of our current and previous studies indicate that the actions of aromatized testosterone, which signals via ERα, are required for defeminization of the AVPV and masculinization of the BNSTp. Neonatal treatment with a selective agonist for ERα or ERβ increases the volume and neuron number of the BNSTp in female mice, but does not completely masculinize the BNSTp [22]. Accordingly, it appears that estradiol binding to both ERα and ERβ during the postnatal period is necessary to masculinize the BNSTp. However, we previously showed that the BNSTp of βERKO mice is masculinized normally, although masculinization of the BNSTp is disrupted in αERKO mice [23]. Based on the results of our previous study using βERKO mice, ERβ may not be necessary for masculinization of the BNSTp.

We further examined the AVPV and BNSTp of ARKO mice. Consequently, we found that masculinization of the BNSTp was disrupted by AR gene deletion, although it appeared that defenimization of the AVPV proceeded normally without the AR gene. Adult ARKO male mice exhibit testicular atrophy resulting in a decreased level of serum testosterone [33]. The serum testosterone level of newborn ARKO male mice is also lower than that of newborn WT males [34]. In this context, the abnormality observed in ARKO male mice may be due not only to the lack of the AR gene itself, but also to decreased production of testosterone. We therefore injected TP, an aromatizable androgen, into newborn ARKO male mice to compensate for decreased production of testosterone during the postnatal period and analyzed the morphology of the resulting BNSTp. As a result, we did not find any significant effect of TP, and the dose and injection time of TP were sufficient to masculinize the volume and number of neurons in the BNSTp of female mice [22]. This finding indicated that disrupting masculinization of the BNSTp in ARKO males was mainly due to AR gene deletion and not a reduction in testosterone levels during the postnatal period. Thus, masculinization of the BNSTp may involve not only the action of aromatized testosterone binding to ERα, but also the action of testosterone binding to AR. On the other hand, the action of aromatized testosterone binding to ERα may be sufficient to defeminize the AVPV.

Previous studies have indicated that defeminization of the number of TH-expressing neurons in the AVPV is caused by the actions of estradiol binding to ERα and ERβ, but not the action of testosterone binding to AR [27], [28]. However, we did not find a significant effect of ERβ gene deletion on the total neuron number in the AVPV of mice. The effect of ERβ gene deletion on TH-expressing neurons in the AVPV may be masked in the results of our study, because TH-expressing neurons are a small proportion of the total neuronal population in the AVPV. In rats, the total neuron number in the AVPV of females is decreased by neonatal treatment with selective agonists for ERα and for ERβ [29], indicating that both ERα and ERβ are required for defeminization of the rat AVPV. There may be differences between species in terms of the actions of sex steroids on the formation of the AVPV.

### Sex steroid actions on the AVPV and BNSTp in perinatal mice and the regional difference

We next examined the gene expression of aromatase, ERα, ERβ, and AR in the AVPV and BNSTp of WT and ARKO mice on ED18 and PD4 that are in the classically identified critical period when testicular testosterone effectively exhibits organizational effects on sexual differentiation of the rodent brain [20]. In terms of aromatase and ERα, which are necessary to defeminize the AVPV, the expression of these genes in the AVPV did not differ between sexes and was unaffected by AR gene deletion. In addition, aromatase expression in the AVPV was higher on ED18, whereas ERα expression was higher on PD4. Testicular testosterone production during the perinatal period of mice reaches a peak on ED18, and testosterone production in the testes on PD4 is lower than that on ED18 [36]. Therefore, higher aromatase expression on ED18 may indicate that local synthesis of estradiol in the AVPV is more active on ED18 than on PD4. In this study, we did not observe expression of AR or ERβ in the AVPV of ED18 mice. These results support the idea that testicular testosterone mainly exerts its actions in the AVPV during the late fetal period via binding to ERα after it is locally converted to estradiol by aromatase. This action may be involved in defeminization of the AVPV.

In addition to the AVPV of WT mice, AR expression in the BNSTp of WT mice was not detected on ED18, although AR was highly expressed on PD4. This result is consistent with a previous study showing that AR is not expressed in the BNSTp of fetal mice, even though AR is found in the BNSTp of neonatal mice [39]. These findings indicate that testosterone signaling via AR in the BNSTp is not functional during the prenatal period, despite higher testosterone production in the testes during the prenatal period than the postnatal period [36]. Testosterone signaling via AR after birth may be involved in masculinization of the BNSTp. However, there is a report showing that neonatal treatment with dihydrotestosterone propionate, a non-aromatizable androgen, has no masculinizing effect on the BNSTp in female mice [22]. A reasonable explanation for the involvement of AR on masculinization of the BNSTp is that the direct actions of testicular testosterone through binding to AR are exerted on the BNSTp during prepubertal and/or pubertal periods. It was recently reported that the sex difference in the neuron number of the BNSTp becomes marked in adulthood with an increasing neuron number in the male BNSTp and loss of neurons in the female BNSTp at 20 days after birth [40]. This previous report suggests that ovarian and testicular hormones during prepubertal and pubertal periods act in demasculinization and masculinization of the BNSTp, respectively. In this context, in the case of ARKO male mice, we are not able to exclude the possibility that decreased levels of testosterone during pre-puberty and puberty, which are caused by testicular atrophy, is at least partly responsible for disruption of masculinization in the BNSTp.

Another explanation for the involvement of AR in masculinization of the BNSTp is that testosterone binding to AR modifies the actions of aromatized testosterone via ERα for masculinization of the BNSTp during the postnatal period. In this study, we found that deletion of the AR gene resulted in a significant increase of aromatase and ERα expression in the BNSTp of male mice on PD4. These results suggest that testosterone acts via AR to suppress aromatase and ERα expression in the BNSTp of postnatal males, resulting in attenuation of aromatized testosterone actions via ERα. Our previous study indicated that estradiol signaling via ERα during the postnatal period plays an essential role in masculinization of the BNSTp [23]. However, ovarian hormones, mainly estradiol, during puberty may act to demasculinize the BNSTp in female mice, because the number of calbindin-expressing neurons in the female BNSTp decreases significantly between pre-puberty and adolescence [40]. It appears likely that estradiol has dual effects on the formation of the BNSTp. Estradiol during the perinatal period has masculinizing effects in male mice, whereas estradiol during puberty has demasculinizing effects in female mice. To achieve successful formation of the male BNSTp, testicular testosterone during the prenatal period may be converted mostly to estradiol in the BNSTp, which exerts its actions to masculinize the BNSTp. After birth, estradiol synthesis and signaling via ERα may be decreased gradually as testosterone signals via AR. Alternatively, direct actions of testosterone though binding to AR may be enhanced after birth. Testosterone masculinizes and defeminizes sexual behavioral patterns in male hamsters during the prepubertal period [16], [41]. However, EB treatment from PD16 to PD26 has no effect on female-directed mounting behavior in testosterone-treated ovariectomized mice in adulthood, although EB treatment from PD6 to PD16 increases such behavior [42]. Thus, during late postnatal and prepubertal periods, testicular testosterone itself may participate in formation of the male brain.

In conclusion, we show that aromatase and ERα are required to defeminize the AVPV in mice, while ERβ and AR are unnecessary. On the other hand, our previous and current studies indicate that masculinization of the BNSTp involves aromatase, ERα, and AR, but not ERβ [23]. We also found that the expression patterns of aromatase and ERα genes differ between the AVPV and BNSTp during the perinatal period. Moreover, testosterone signaling via AR during the postnatal period inhibits the expression of aromatase and ERα in the BNSTp, but not the AVPV. Taken together, there appears to be a regional difference in sex steroid actions on the formation of morphological sex differences in the AVPV and BNSTp. Both estradiol signaling via ERα during the perinatal period and testosterone signaling via AR during the postnatal period may be required for masculinization of the BNSTp, whereas the former may be sufficient for defeminization of the AVPV.

## Supporting Information

Figure S1 **Mating procedure to obtain ARKO offspring.** Heterozygous ARKO female mice expressing Cre recombinase (Cre AR^-/+^) were mated with male mice with a floxed AR gene (AR^flox/Y^), resulting in the generation of offspring with four different genotypes in each sex: Cre AR^-/Y^ (CreKO), Cre AR^+/Y^ (CreWT), AR^-/Y^ (KO), and AR^+/Y^ (WT) in male offspring, and Cre AR^-/-^ (CreKO), Cre AR^-/+^ (CreHZ), AR^flox/-^ (FloxHZ), and AR^flox/+^ (FloxWT) in female offspring. In this study, we used CreWT and WT males as WT males, CreKO and KO males as ARKO males, FloxWT females as WT females, and CreKO females as ARKO females.
(TIF)

Figure S2 **Representative photomicrographs of cresyl fast violet-stained cells in the brain.** (**A**) Neuronal cells, (**B**) glial cells, and (**C**) endothelial cells. Scale bars indicate 5 µm.
(TIF)

Table S1 **Stereological analyses of neuronal and glial cells in the AVPV of AromKO mice.**
(DOCX)

Table S2 **Stereological analyses of neuronal and glial cells in the AVPV of αERKO mice.**
(DOCX)

Table S3 **Stereological analyses of neuronal and glial cells in the AVPV of βERKO mice.**
(DOCX)

Table S4 **Stereological analyses of neuronal and glial cells in the AVPV of ARKO mice.**
(DOCX)

Table S5 **Stereological analyses of neuronal and glial cells in the BNSTp of ARKO mice.**
(DOCX)

Table S6 **Stereological analyses of neuronal and glial cells in the BNSTp of TP-treated ARKO mice.**
(DOCX)
