# Supplementary figures and images for: Regional Difference in Sex Steroid Action on Formation of Morphological Sex Differences in the Anteroventral Periventricular Nucleus and Principal Nucleus of the Bed Nucleus of the Stria Terminalis
Source: PLoS One. 2014 Nov 14;9(11):e112616. doi: 10.1371/journal.pone.0112616 (PMC4232352; doi:10.1371/journal.pone.0112616)

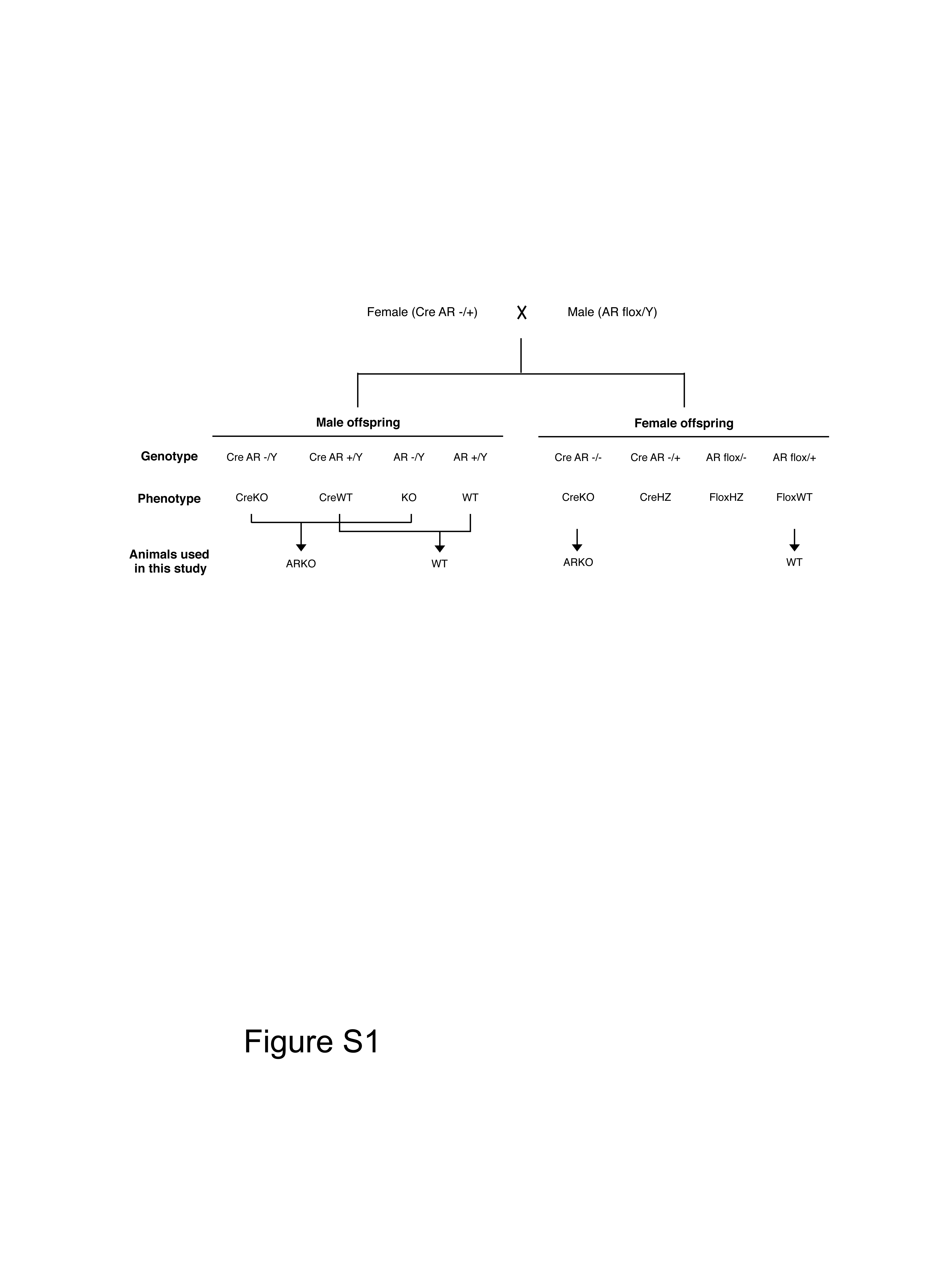

Supplement: Figure S1 — Mating procedure to obtain ARKO offspring. Heterozygous ARKO female mice expressing Cre recombinase (Cre AR-/+) were mated with male mice with a floxed AR gene (ARflox/Y), resulting in the generation of offspring with four different genotypes in each sex: Cre AR-/Y (CreKO), Cre AR+/Y (CreWT), AR-/Y (KO), and AR+/Y (WT) in male offspring, and Cre AR-/- (CreKO), Cre AR-/+ (CreHZ), ARflox/- (FloxHZ), and ARflox/+ (FloxWT) in female offspring. In this study, we used CreWT and WT males as WT males, CreKO and KO males as ARKO males, FloxWT females as WT females, and CreKO females as ARKO females. (TIF) [file pone.0112616.s001.tif]

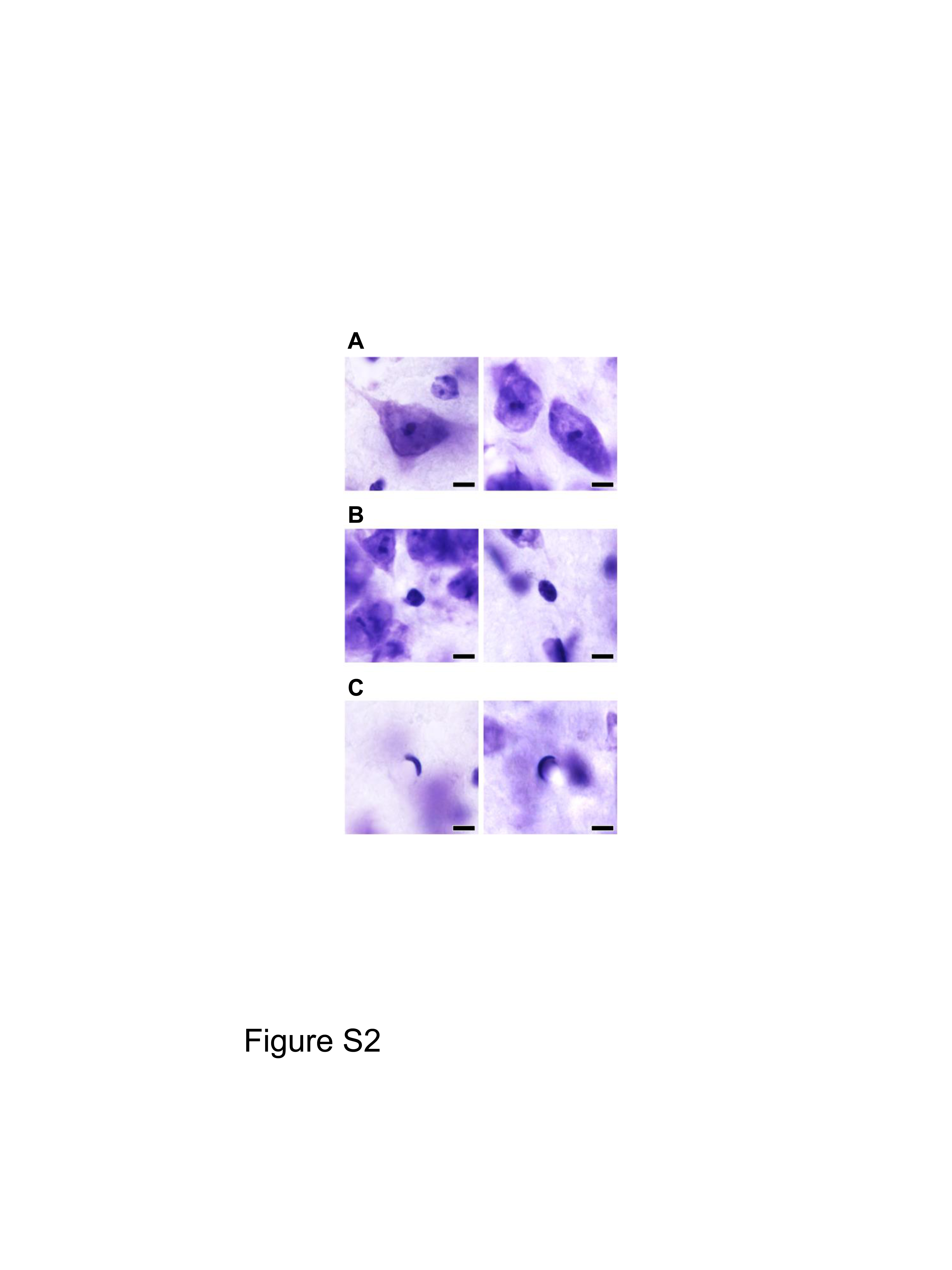

Supplement: Figure S2 — Representative photomicrographs of cresyl fast violet-stained cells in the brain. (A) Neuronal cells, (B) glial cells, and (C) endothelial cells. Scale bars indicate 5 µm. (TIF) [file pone.0112616.s002.tif]
