# Supplementary material for: Regional Difference in Sex Steroid Action on Formation of Morphological Sex Differences in the Anteroventral Periventricular Nucleus and Principal Nucleus of the Bed Nucleus of the Stria Terminalis
Source: PLoS One. 2014 Nov 14;9(11):e112616. doi: 10.1371/journal.pone.0112616 (PMC4232352; doi:10.1371/journal.pone.0112616)
Supplement: Table S1 — Stereological analyses of neuronal and glial cells in the AVPV of AromKO mice. (DOCX) [file pone.0112616.s003.docx]

**Table S1. Stereological analyses of neuronal and glial cells in the AVPV of AromKO mice.**

|  | WT male (n = 5) | AromKO male (n = 5) | WT female (n = 5) | AromKO female (n = 5) |
| --- | --- | --- | --- | --- |
| No. of sections | 3.00 ± 0.00 | 4.00 ± 0.00 | 3.80 ± 0.20 | 3.60 ± 0.24 |
| No. of sampling sites | 16.40 ± 0.98 | 24.00 ± 1.38 | 22.80 ± 1.71 | 23.00 ± 2.12 |
| Total number of neuronal cells counted | 43.00 ± 1.00 | 74.40 ± 6.06 | 68.40 ± 7.84 | 81.60 ± 9.69 |
| Total number of neuronal cells estimated | 5267.50 ± 122.50 | 9114.00 ± 742.72 | 8379.00 ± 960.36 | 9996.00 ± 1186.80 |
| Neuron density (number/mm^3^) × 10^−4^ * | 3.08 ± 0.05 | 3.59 ± 0.19 | 3.61 ± 0.26 | 4.45 ± 0.29 |
| Coefficient of error (Shmitz-Hof) of neurons | 0.15 ± 0.0018 | 0.12 ± 0.0053 | 0.12 ± 0.0081 | 0.11 ± 0.0085 |
| Total number of glial cells counted | 3.60 ± 0.68 | 6.60 ± 1.17 | 3.40 ± 0.60 | 3.80 ± 0.20 |
| Total number of glial cells estimated ** | 441.00 ± 83.08 | 808.50 ± 142.86 | 416.50 ± 73.50 | 465.50 ± 24.50 |
| Glial cell density (number/mm^3^) × 10^−5^ | 2.56 ± 0.45 | 3.19 ± 0.55 | 1.86 ± 0.43 | 2.16 ± 0.22 |
| Coefficient of error (Shmitz-Hof) of glial cells | 0.55 ± 0.049 | 0.43 ± 0.029 | 0.57 ± 0.056 | 0.56 ± 0.015 |

Common parameters: section thickness: 30 μm; section interval: 60 μm; sampling grid size: 140 × 140 μm; counting frame size: 20 × 20 μm; dissector height: 12 μm; guard zone height: 2 μm.

*, Significant effect of sex (p < 0.01) and genotype (p < 0.01).

**, Significant effect of genotype (p < 0.05).
