# Supplementary material for: Regional Difference in Sex Steroid Action on Formation of Morphological Sex Differences in the Anteroventral Periventricular Nucleus and Principal Nucleus of the Bed Nucleus of the Stria Terminalis
Source: PLoS One. 2014 Nov 14;9(11):e112616. doi: 10.1371/journal.pone.0112616 (PMC4232352; doi:10.1371/journal.pone.0112616)
Supplement: Table S2 — Stereological analyses of neuronal and glial cells in the AVPV of αERKO mice. (DOCX) [file pone.0112616.s004.docx]

**Table S2. Stereological analyses of neuronal and glial cells in the AVPV of αERKO mice.**

|  | WT male (n = 4) | αERKO male (n = 3) | WT female (n = 5) | αERKO female (n = 5) |
| --- | --- | --- | --- | --- |
| No. of sections | 3.00 ± 0.00 | 4.33 ± 0.33 | 4.00 ± 0.00 | 3.60 ± 0.24 |
| No. of sampling sites | 15.25 ± 0.85 | 24.67 ± 4.91 | 22.00 ± 2.41 | 22.40 ± 2.42 |
| Total number of neuronal cells counted | 46.25 ± 3.42 | 80.33 ± 6.36 | 77.00 ± 9.72 | 73.80 ± 5.58 |
| Total number of neuronal cells estimated | 5665.63 ± 419.54 | 10404.33 ± 377.00 | 9746.10 ± 1124.82 | 9731.40 ± 721.12 |
| Neuron density (number/mm^3^) × 10^−4^ | 3.77 ± 0.38 | 3.83 ± 0.45 | 4.38 ± 0.24 | 4.46 ± 0.23 |
| Coefficient of error (Shmitz-Hof) of neurons | 0.15 ± 0.0050 | 0.11 ± 0.0044 | 0.12 ± 0.0062 | 0.12 ± 0.0044 |
| Total number of glial cells counted | 4.00 ± 1.00 | 10.00 ± 1.00 | 9.20 ± 1.46 | 8.00 ± 1.38 |
| Total number of glial cells estimated | 490.05 ± 122.48 | 1314.83 ± 184.61 | 1195.60 ± 238.72 | 1087.80 ± 229.52 |
| Glial cell density (number/mm^3^) × 10^−5^ | 3.21 ± 0.74 | 5.03 ± 1.34 | 5.52 ± 1.27 | 5.17 ± 0.12 |
| Coefficient of error (Shmitz-Hof) of glial cells | 0.53 ± 0.050 | 0.32 ± 0.017 | 0.34 ± 0.030 | 0.37 ± 0.037 |

Common parameters: section thickness: 30 μm; section interval: 60 μm; sampling grid size: 140 × 140 μm; counting frame size: 20 × 20 μm; dissector height: 12 μm; guard zone height: 2 μm.
