# Supplementary material for: Regional Difference in Sex Steroid Action on Formation of Morphological Sex Differences in the Anteroventral Periventricular Nucleus and Principal Nucleus of the Bed Nucleus of the Stria Terminalis
Source: PLoS One. 2014 Nov 14;9(11):e112616. doi: 10.1371/journal.pone.0112616 (PMC4232352; doi:10.1371/journal.pone.0112616)
Supplement: Table S3 — Stereological analyses of neuronal and glial cells in the AVPV of βERKO mice. (DOCX) [file pone.0112616.s005.docx]

**Table S3. Stereological analyses of neuronal and glial cells in the AVPV of βERKO mice.**

|  | WT male (n = 5) | βERKO male (n = 5) | WT female (n = 5) | βERKO female (n = 5) |
| --- | --- | --- | --- | --- |
| No. of sections | 3.20 ± 0.20 | 3.40 ± 0.24 | 4.20 ± 0.20 | 3.80 ± 0.20 |
| No. of sampling sites | 13.80 ± 1.16 | 16.60 ± 1.50 | 23.20 ± 1.59 | 19.20 ± 1.85 |
| Total number of neuronal cells counted | 47.60 ± 3.33 | 57.00 ± 5.07 | 86.20 ± 2.85 | 79.40 ± 8.00 |
| Total number of neuronal cells estimated | 5736.27 ± 338.52 | 6835.17 ± 626.82 | 10559.40 ± 349.48 | 9569.70 ± 964.76 |
| Neuron density (number/mm^3^) × 10^−4^ | 4.20 ± 0.42 | 4.47 ± 0.38 | 4.63 ± 0.61 | 0.51 ± 0.63 |
| Coefficient of error (Shmitz-Hof) of neurons | 0.15 ± 0.0050 | 0.14 ± 0.0106 | 0.11 ± 0.0017 | 0.11 ± 0.0061 |
| Total number of glial cells counted | 3.00 ± 1.44 | 4.40 ± 2.29 | 2.80 ± 0.97 | 2.80 ± 1.16 |
| Total number of glial cells estimated | 367.50 ± 139.67 | 537.04 ± 281.47 | 343.00 ± 118.77 | 342.02 ± 142.19 |
| Glial cell density (number/mm^3^) × 10^−5^ | 2.50 ± 0.94 | 2.96 ± 1.30 | 1.33 ± 0.37 | 1.57 ± 0.5 |
| Coefficient of error (Shmitz-Hof) of glial cells | 0.61 ± 0.14 | 0.53 ± 0.10 | 0.72 ± 0.12 | 0.64 ± 0.14 |

Common parameters: section thickness: 30 μm; section interval: 60 μm; sampling grid size: 140 × 140 μm; counting frame size: 20 × 20 μm; dissector height: 12 μm; guard zone height: 2 μm.
