# Supplementary material for: Regional Difference in Sex Steroid Action on Formation of Morphological Sex Differences in the Anteroventral Periventricular Nucleus and Principal Nucleus of the Bed Nucleus of the Stria Terminalis
Source: PLoS One. 2014 Nov 14;9(11):e112616. doi: 10.1371/journal.pone.0112616 (PMC4232352; doi:10.1371/journal.pone.0112616)
Supplement: Table S4 — Stereological analyses of neuronal and glial cells in the AVPV of ARKO mice. (DOCX) [file pone.0112616.s006.docx]

**Table S4. Stereological analyses of neuronal and glial cells in the AVPV of ARKO mice.**

|  | WT male (n = 9) | ARKO male (n = 10) | WT female (n = 4) | ARKO female (n = 4) |
| --- | --- | --- | --- | --- |
| No. of sections | 3.78 ± 0.15 | 3.80 ± 0.13 | 4.50 ± 0.29 | 4.75 ± 0.25 |
| No. of sampling sites | 15.67 ± 1.38 | 15.40 ± 1.01 | 26.25 ± 1.25 | 28.00 ± 0.71 |
| Total number of neuronal cells counted | 40.11 ± 3.53 | 45.00 ± 4.02 | 82.00 ± 9.55 | 76.25 ± 6.30 |
| Total number of neuronal cells estimated | 4913.72 ± 432.86 | 5512.50 ± 493.05 | 10045.00 ± 1169.65 | 9340.63 ± 772.13 |
| Neuron density (number/mm^3^) × 10^−4^ | 3.18 ± 0.22 | 3.44 ± 0.25 | 3.96 ± 0.67 | 3.47 ± 0.20 |
| Coefficient of error (Shmitz-Hof) of neurons | 0.16 ± 0.0076 | 0.15 ± 0.0072 | 0.11 ± 0.0069 | 0.12 ± 0.0048 |
| Total number of glial cells counted | 4.11 ± 0.75 | 3.70 ± 0.73 | 5.25 ± 0.63 | 7.50 ± 1.26 |
| Total number of glial cells estimated * | 503.61 ± 92.32 | 453.25 ± 89.55 | 643.13 ± 77.07 | 918.75 ± 154.14 |
| Glial cell density (number/mm^3^) × 10^−5^ | 3.28 ± 0.63 | 2.61 ± 0.47 | 2.57 ± 0.53 | 3.36 ± 0.41 |
| Coefficient of error (Shmitz-Hof) of glial cells | 0.57 ± 0.065 | 0.61 ± 0.074 | 0.44 ± 0.025 | 0.38 ± 0.030 |

Common parameters: section thickness: 30 μm; section interval: 60 μm; sampling grid size: 140 × 140 μm; counting frame size: 20 × 20 μm; dissector height: 12 μm; guard zone height: 2 μm.

*, Significant effect of sex (p < 0.01).
