# Supplementary material for: Regional Difference in Sex Steroid Action on Formation of Morphological Sex Differences in the Anteroventral Periventricular Nucleus and Principal Nucleus of the Bed Nucleus of the Stria Terminalis
Source: PLoS One. 2014 Nov 14;9(11):e112616. doi: 10.1371/journal.pone.0112616 (PMC4232352; doi:10.1371/journal.pone.0112616)
Supplement: Table S5 — Stereological analyses of neuronal and glial cells in the BNSTp of ARKO mice. (DOCX) [file pone.0112616.s007.docx]

**Table S5. Stereological analyses of neuronal and glial cells in the BNSTp of ARKO mice.**

|  | WT male (n = 9) | ARKO male (n = 10) | WT female (n = 4) | ARKO female (n = 4) |
| --- | --- | --- | --- | --- |
| No. of sections | 5.56 ± 0.24 | 5.00 ± 0.37 | 5.00 ± 0.41 | 4.50 ± 0.29 |
| No. of sampling sites | 23.78 ± 1.16 | 17.00 ± 0.97 | 13.75 ± 1.93 | 14.00 ± 1.78 |
| Total number of neuronal cells counted | 56.78 ± 2.86 | 38.30 ± 3.28 | 33.00 ± 5.18 | 32.50 ± 3.18 |
| Total number of neuronal cells estimated | 14194.44 ± 714.16 | 9621.43 ± 800.19 | 8380.68 ± 1215.05 | 8125.00 ± 793.86 |
| Neuron density (number/mm^3^) × 10^−4^ | 3.00 ± 0.19 | 3.07 ± 0.25 | 2.79 ± 0.40 | 2.71 ± 0.25 |
| Coefficient of error (Shmitz-Hof) of neurons | 0.13 ± 0.0035 | 0.17 ± 0.0071 | 0.18 ± 0.013 | 0.18 ± 0.0095 |
| Total number of grail cells counted | 5.67 ± 1.15 | 3.70 ± 0.54 | 5.25 ± 1.93 | 3.50 ± 0.65 |
| Total number of grail cells estimated | 1416.67 ± 288.68 | 937.48 ± 141.79 | 1335.23 ± 478.39 | 875.00 ± 161.37 |
| Glial cell density (number/mm^3^) × 10^−5^ | 2.90 ± 0.51 | 2.89 ± 0.40 | 4.28 ± 1.34 | 2.84 ± 0.37 |
| Coefficient of error (Shmitz-Hof) of glial cells | 0.50 ± 0.070 | 0.59 ± 0.070 | 0.49 ± 0.065 | 0.56 ± 0.056 |

Common parameters: section thickness: 30 μm; section interval: 60 μm; sampling grid size: 200 × 200 μm; counting frame size: 20 × 20 μm; dissector height: 12 μm; guard zone height: 2 μm.
