# Supplementary material for: Regional Difference in Sex Steroid Action on Formation of Morphological Sex Differences in the Anteroventral Periventricular Nucleus and Principal Nucleus of the Bed Nucleus of the Stria Terminalis
Source: PLoS One. 2014 Nov 14;9(11):e112616. doi: 10.1371/journal.pone.0112616 (PMC4232352; doi:10.1371/journal.pone.0112616)
Supplement: Table S6 — Stereological analyses of neuronal and glial cells in the BNSTp of TP-treated ARKO mice. (DOCX) [file pone.0112616.s008.docx]

**Table S6. Stereological analyses of neuronal and glial cells in the BNSTp of TP-treated ARKO mice.**

|  | WT + oil (n = 4) | WT + TP (n = 6) | ARKO + oil (n = 4) | ARKO + TP (n = 6) |
| --- | --- | --- | --- | --- |
| No. of sections | 3.75 ± 0.25 | 3.67 ± 0.21 | 3.50 ± 0.29 | 3.67 ± 0.21 |
| No. of sampling sites | 30.25 ± 1.80 | 27.67 ± 1.28 | 23.00 ± 1.22 | 23.50 ± 1.45 |
| Total number of neuronal cells counted | 75.00 ± 4.64 | 70.50 ± 3.83 | 55.00 ± 3.54 | 57.50 ± 3.27 |
| Total number of neuronal cells estimated | 18750.00 ± 1159.20 | 17625.00 ± 956.88 | 13750.00 ± 883.88 | 14375.00 ± 818.41 |
| Neuron density (number/mm^3^) × 10^−4^ | 3.22 ± 0.21 | 3.17 ± 0.19 | 2.71 ± 0.04 | 3.07 ± 0.18 |
| Coefficient of error (Shmitz-Hof) of neurons | 0.12 ± 0.0038 | 0.12 ± 0.0036 | 0.14 ± 0.0047 | 0.13 ± 0.0036 |
| Total number of grail cells counted | 7.00 ± 0.91 | 5.67 ± 1.26 | 7.25 ± 2.02 | 5.83 ± 0.65 |
| Total number of grail cells estimated | 1750.00 ± 228.22 | 1416.67 ± 314.02 | 1812.50 ± 503.89 | 1458.33 ± 163.51 |
| Glial cell density (number/mm^3^) × 10^−5^ | 2.97 ± 0.31 | 2.50 ± 0.53 | 3.62 ± 1.02 | 3.11 ± 0.35 |
| Coefficient of error (Shmitz-Hof) of glial cells | 0.39 ± 0.026 | 0.50 ± 0.103 | 0.40 ± 0.048 | 0.43 ± 0.025 |

Common parameters: section thickness: 30 μm; section interval: 60 μm; sampling grid size: 200 × 200 μm; counting frame size: 20 × 20 μm; dissector height: 12 μm; guard zone height: 2–3 μm.
